# Supplementary material for: Lentils and Yeast Fibers: A New Strategy to Mitigate Enterotoxigenic Escherichia coli (ETEC) Strain H10407 Virulence?
Source: Nutrients. 2022 May 21;14(10):2146. doi: 10.3390/nu14102146 (PMC9144138; doi:10.3390/nu14102146)
Supplement: Supplementary file 1 [file nutrients-14-02146-s001.zip › nutrients-1701941-supplementary.pdf]

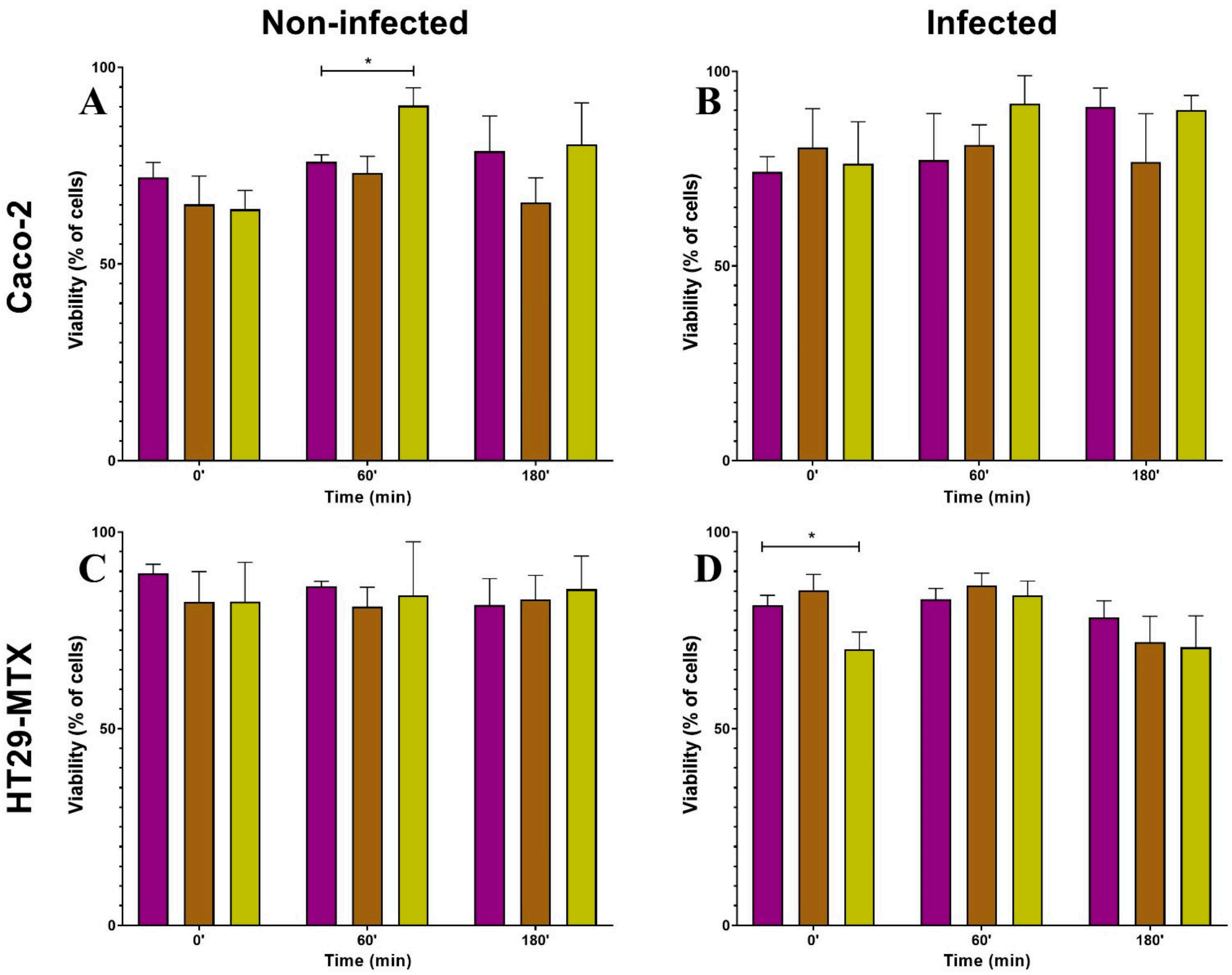

Figure S1. Effect of dietary-fiber products and ETEC H10407 infection on intestinal cell viability. Cell medium with or without dietary-fiber-containing products (lentils or yeast, 2 g.L^{-1}) was added on the apical side of the transwell of Caco-2 (**A**,**B**) and HT29-MTX (**C**,**D**) cells and infected (**B**,**D**) or not with ETEC strain H10407 (10^7 CFU.mL^{-1}) (**A**,**C**). Cell viability was then analyzed for 3 h using a Trypan blue exclusion assay. Graphs present the mean ± SD of three independent experiments. Statistical differences with the control condition (not infected, non-treated) were found by Dunnett's multiple comparisons (*: $p < 0.05$).

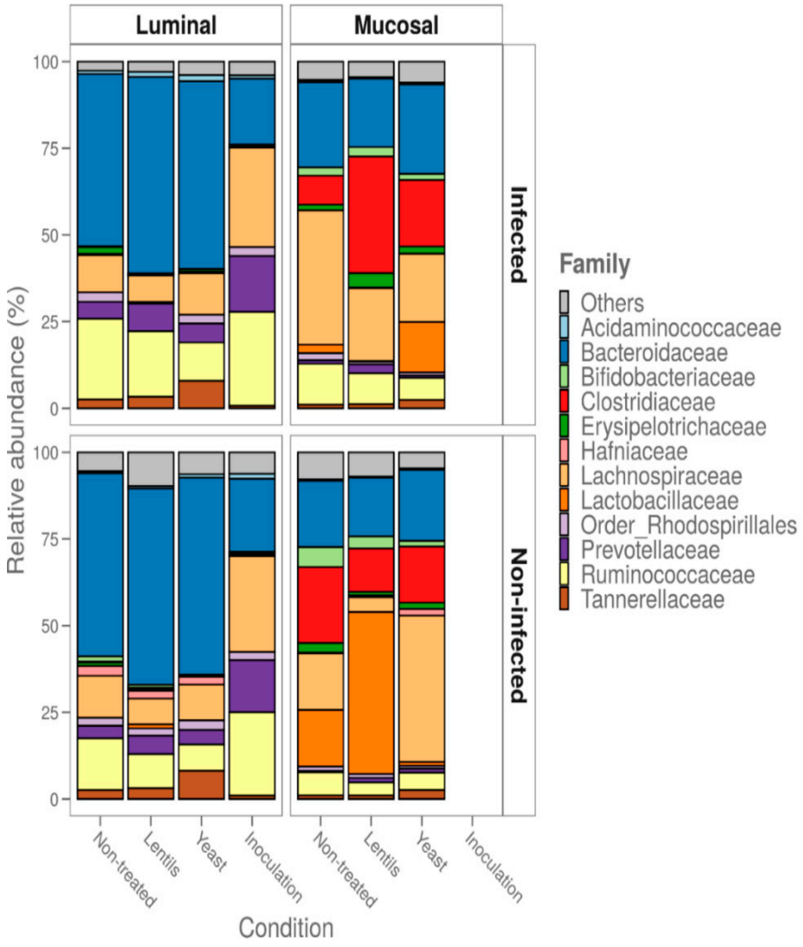

Figure S2. Cumulative bar plots of fiber-containing products and ETEC modulation of microbiota composition at the family level, excluding ASV1. Batch experiments were performed using feces from six healthy donors, challenged or not with ETEC strain H10407, and treated or not with the dietary-fiber-containing products. The graphs show cumulative bar plots of the relative microbial community composition at the family level, excluding ASV1, belonging to *Escherichia*/*Shigella*, which had the highest number of reads. The area graphs show the relative abundance of the 12 most abundant families in all six different donors confounded.

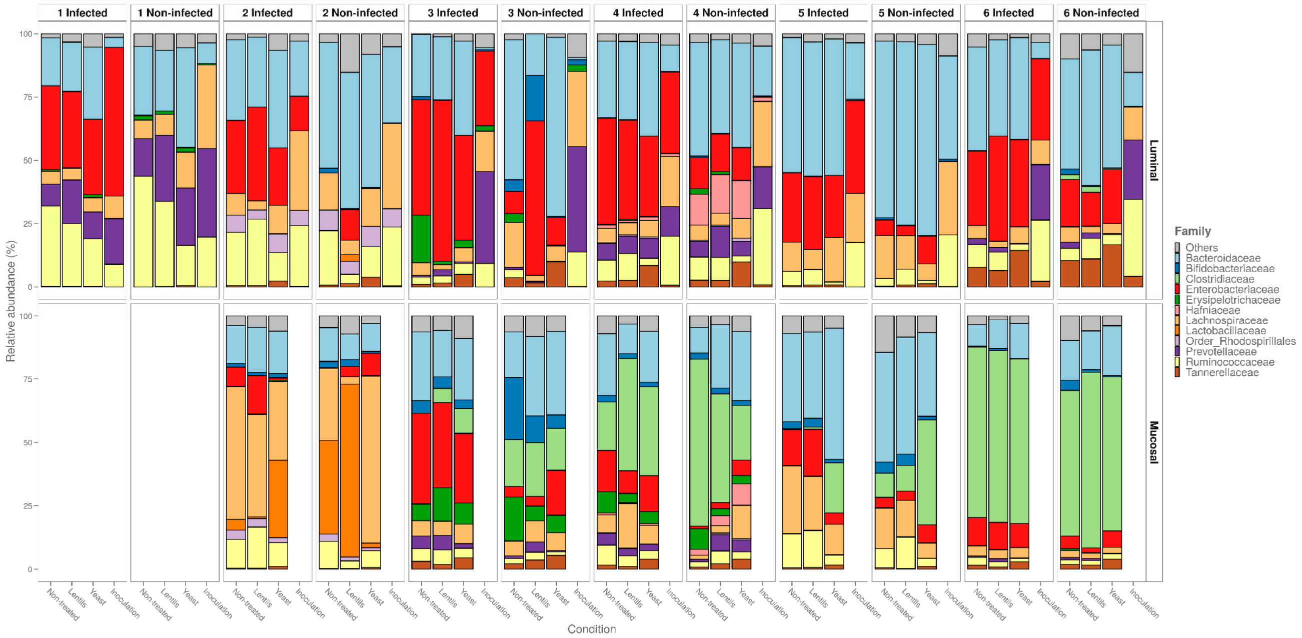

Figure S3. Donor-specific impact of dietary-fiber-containing products on ETEC modulation of microbiota β-diversity at the family level. Cumulative bar plots of the relative microbial community composition in fecal batch experiments at the family level. The graphs show the relative abundances of the 12 most abundant families in the luminal and mucosal phases for the six different donors, as determined by amplicon sequencing. Mucosal phase data are missing for donor 1 due to a technical problem.

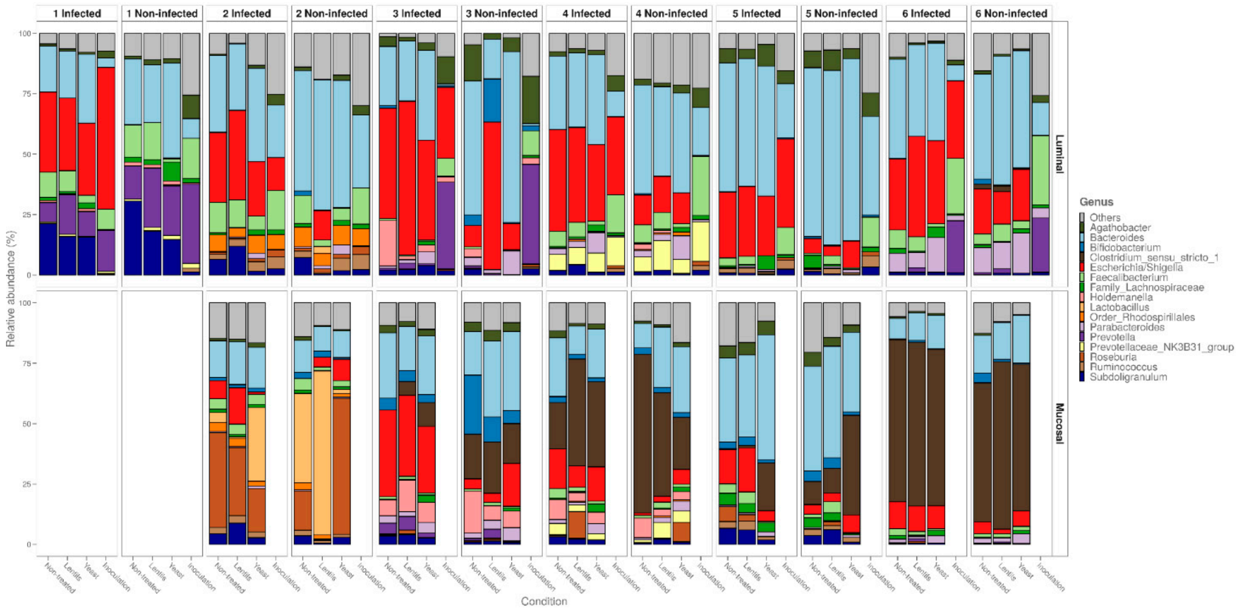

Figure S4. Donor-specific impact of dietary-fiber-containing products on ETEC modulation of microbiota β-diversity at the genus level. Cumulative bar plots of the relative microbial community composition in fecal batch experiments at the genus level. The graphs show the relative abundance of the 16 most abundant genera in the luminal and mucosal phases for the six different donors, as determined by amplicon sequencing. Mucosal phase data are missing for donor 1 due to a technical problem.
